# Competing risk model for prognostic comparison between clear cell type and common type hepatocellular carcinoma: A population‐based propensity score matching study

**DOI:** 10.1002/cam4.5773

**Published:** 2023-03-19

**Authors:** Xiao Zhong, Xingwang Hu, Xue‐Gong Fan

**Affiliations:** ^1^ Department of Infectious Diseases, Hunan Key Laboratory of Viral Hepatitis, Xiangya Hospital Central South University Changsha China

**Keywords:** clear cell carcinoma, competing risk model, hepatocellular carcinoma

## Abstract

**Background:**

Clear cell type hepatocellular carcinoma (HCC) is an uncommon neoplasm with an ambivalent prognosis compared to common type HCC.

**Methods:**

First, patients with clear cell or common type HCC were enrolled from the Surveillance, Epidemiology, and End Results (SEER) database, and their demographic and clinical characteristics were identified. Next, overall survival (OS), disease‐specific survival (DSS), and subgroup analysis of the two types of HCC were performed. Next, we utilized a competing risk model to focus on cancer‐caused death. Finally, propensity score matching (PSM) was employed to reduce the confounding factors based on the histopathological type, and sensitivity analysis was conducted.

**Results:**

A total of 205 cases of clear cell type HCC and 29,954 cases of common type HCC were enrolled in our study. Patients with clear cell type HCC were older and predominantly female than those with common type HCC. OS and DSS were not significantly different between the two groups, and histopathological type was not a prognostic factor of HCC, as verified by the competing risk model. Patient characteristics adjusted by PSM and sensitivity analysis confirmed this conclusion. In subgroup analysis, patients with clear cell type HCC at grade III ~ IV and with lymph nodes metastasis had a better prognosis compared to common type HCC.

**Conclusions:**

This study revealed that the prognosis of clear cell type HCC is similar to common type HCC. Tumor differentiation grade and status of lymph node metastasis affect the prognosis of HCC.

## Graphical abstract

Patients with HCC‐clear and HCC‐NOS were enrolled from the SEER database to identify demographic and clinical characteristics. Prognostic analyses were performed using OS, DSS, multivariate regression analysis, subgroup analysis, etc. Competing risk model and PSM were introduced for reducing confounding factors. Sensitivity analysis was included for conclusion validation.

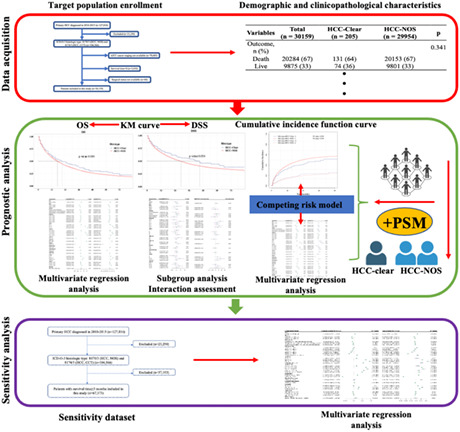

## 1 INTRODUCTION

The most common type of primary liver cancer is hepatocellular carcinoma (HCC). Primary liver cancer ranks as the third highest cause of cancer‐related death and the sixth highest‐incidence cancer worldwide. The incidence and mortality of HCC are highest in East Asia and Africa.^1^, ^2^ Moreover, the mortality rate worldwide of HCC is still increasing.^3^, ^4^ In the 4th edition of WHO classification of the tumors of the digestive system published in 2010, cytologically, an uncommon variant of clear cell type HCC was included.^5^ Prior to this, clear cell carcinoma was broadly understood to uniquely belong to renal or ovarian cancer owing to their high incidence. In fact, clear cell type HCC is a particular form of well‐differentiated HCC, accounting for 0.4%–37% of all hepatocellular carcinomas.^6^, ^7^, ^8^, ^9^ With hematoxylin–eosin staining, in the clearing cytoplasm of clear cell type HCC, glycogen was mild to marked accumulated while fat was less frequently stored.^10^ To diagnose the clear cell type HCC, the neoplastic cells filled with clearing cytoplasm are necessary, while the percentage of this kind of neoplastic cells varies based on different literature.^7^, ^11^, ^12^ The cutoff of a minimum of 50% was acknowledged in the recent AFIP fascicle.^13^ Hepatic resection was considered the most therapeutic method in clear cell type HCC.^14^ However, few studied have related to survival and prognostic factors, and it remains controversial when discussing better or worse outcomes compared with common type HCC.^7^, ^11^, ^15^, ^16^ Additionally, given the rarity of clear cell type HCC, most associated studies were case reports or had only small sample sizes.

For the first time, our study aimed to discuss the prognostic comparison between patients with clear cell type HCC and normal type HCC. Patients were enrolled from the Surveillance, Epidemiology, and End Results (SEER) database, and confounding factors were regulated by creating a competing risk model and propensity score matching (PSM). Furthermore, our study was examined by performing sensitivity analysis through different datasets to evaluate the credibility of our conclusion. The current study will provide information on the survival and prognosis of clear cell type HCC based on a large‐scale study, thereby assisting hepatic surgeons in treatment decision‐making for patients with clear cell type HCC.

## 2 METHODS

### 2.1 Study population

**FIGURE 1:**
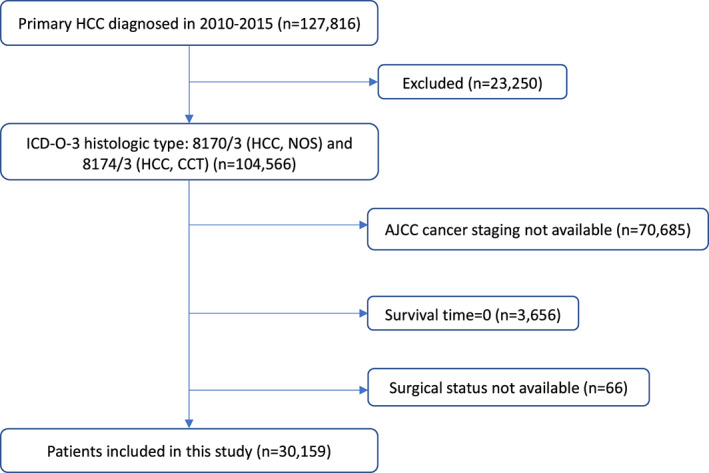
Flow chart of target population selection. The original data were collected from the SEER database. AJCC, American Joint Committee on Cancer; ICD‐0‐3, The International Classification of Diseases for Oncology, third edition; SEER, Surveillance, Epidemiology, and End Results.

The SEER database is an authoritative resource providing cancer statistics with detailed information from the United States National Cancer Institute.^17^ Data from the target population were collected from the SEER database in this study. As shown in Figure 1, patients with clear cell type and common type hepatocellular carcinoma (HCC) between 2010 and 2015 were recruited for further analysis. The inclusion criteria were as follows: (1) patients diagnosed with HCC between 2010 and 2015; (2) liver is the primary site; (3) histopathological diagnosis of clear cell type or common type, based on the histologic type 8170/3 (HCC, not otherwise specific [NOS])/8174/3 (HCC, clear cell type [CCT]) coded by the International Classification of Diseases for Oncology (ICD‐0‐3)^18^; (4) detailed cancer statistics, for example, age, sex, cause of death and American Joint Committee on Cancer (AJCC) cancer staging.^19^ Patients were excluded from this investigation if: (1) the information on AJCC cancer staging and surgery status was not available; (2) survival time was zero; (3) demographic and clinical data such as race, survival months, marital status, and diagnosis year were not definite. Informed consent and intuitional review were exempted since the SEER database is free to access to the public, and patients' privacy was not intruded. Moreover, the population for the sensitivity analysis was identified as patients with primary clear cell type or common type HCC with a survival time of more than or equal to 3 months (Figure S1A).

### 2.2 Variables and outcomes

Covariates considered in this study were survival (months), overall outcome, disease‐specific outcome, age, race, marital status, year of diagnosis, AJCC cancer staging, metastasis, surgical status, radiotherapy, chemotherapy, cancer history, and grade. All detailed information on variables' definitions was available in the SEER database documentation for analysis (https://seer.cancer.gov/analysis/). Cancer staging was identified according to the AJCC 7th staging system. Metastasis bone/brain/liver/lung was related to distant metastasis to bone/brain/liver/lung from primary HCC. Primary means HCC was the first discovered cancer. Grade refers to the degree of differentiation of cancer cells, in which grades I, II, III, and IV represented well differentiated, moderately differentiated, poorly differentiated, and undifferentiated, respectively.

The primary outcome of our study was overall survival (OS) and disease‐specific survival (DSS). Survival months were calculated by the total months from diagnosis time to the time of death or endpoint of follow‐up. DSS is involved as the death due to HCC here. For the competing risk model, the cause of death was divided into death from HCC and death from other causes.

### 2.3 Statistics

As the continuous variable, age was converted to a categorical variable for future analysis. In our study, the comparisons of demographic and clinicopathological categorical variables between HCC‐clear and HCC‐NOS groups were performed using Pearson's chi‐squared test.

To balance the patient characteristics in this study, PSM based on the histopathological type was utilized. The significant covariates in the comparison of the two groups were as follows: sex, race, marital status, AJCC cancer staging, surgical status, radiotherapy, chemotherapy, cancer history, grade, and age. We performed 1:2 PSM to compare prognosis for patients with HCC‐clear and HCC‐NOS, with the caliper value setting as 0.03. The “nearest” method in the R package “MatchIt” was used.^20^, ^21^

Setting OS and DSS as the outcome measurement, the Kaplan–Meier (KM) curve, multivariate Cox regression analysis, subgroup analysis, and interaction assessment were analyzed before and after PSM. The KM method was applied in plotting OS and DSS curves using the survival package of R software.^22^ The log‐rank test was applied to compare the difference between the two groups. Forest plots were graphed using the “forestplot” package of R.^23^ For multivariate, subgroup, and interaction analysis, hazard ratio (HR) and 95% confidence intervals (CI) were assessed using Cox proportional hazard models. Interaction tests were assessed by likelihood ratio tests. The interaction was tested by adding a multiplicative interaction term.

The plot of cumulative incidence function curve and the construction of multivariate analysis of competing risk model before and after PSM were performed using the “cmprsk” package in R.^24^ Additionally, sub‐distribution hazard ratio (SHR) and 95% CI were calculated in competing risk models and sensitivity analysis. Fine and Gray's test was applied to test the difference between the two groups using a cumulative incidence function curve.

In interaction assessment, when DSS was selected as the outcome of interest, AJCC tumor staging, radiotherapy, and history of cancer were excluded as abnormal variables. Metastasis was only analyzed in the KM curve and cumulative incidence function curve to account for the unreliable effect of collinearity with AJCC tumor staging. To keep the information maximization, the AJCC tumor staging variable was retained with most dummy variables relative to AJCC lymph node and distant metastasis staging in the multivariate analysis based on sensitivity analysis considering the collinearity of the above three variables.

R software was utilized for statistical analysis. *p* < 0.05 was considered statistically significant. *p* interaction value <0.05 was considered a statistically significant interaction.

## 3 RESULTS

### 3.1 Demographic and clinicopathological characteristics of the study population

In this investigation, a total of 205 patients with HCC‐clear and 29,954 patients with HCC‐NOS between 2010 to 2015 were recruited. Among these patients, 88 censored patients (43%) with HCC‐clear died from HCC, and the number in HCC‐NOS was 14,508 (48%). We noticed differences in demographic characteristics between these two groups, especially in age and sex (*p* < 0.001 and *p* = 0.001). Regarding AJCC tumor staging, patients with HCC‐clear occupied more T0–T1 and T4 stage than HCC‐NOS.^19^ Over half the population (54%) of HCC‐clear belongs to the T0–T1 stage, and 6% were classified into the T4 stage, while the numbers in HCC‐NOS were 47% and 3%, respectively. No difference was observed between the two groups in terms of distant metastasis. In addition, when comparing treatment between the two groups, surgery was performed in most patients with HCC‐NOS (72%), while patients with HCC‐clear (65%) tended to undergo chemotherapy (*p* < 0.001). The cancer history and differentiation grade of cancer cells were also significantly different in patients with HCC‐clear compared to HCC‐NOS (*p* < 0.001). The detailed information is shown in Table 1.

**TABLE 1:** Comparison of demographic and clinicopathological characteristics between patients with HCC‐clear and HCC‐NOS.

Variables	Total (*n* = 30,159)	HCC‐Clear (*n* = 205)	HCC‐NOS (*n* = 29,954)	*p*
Outcome, *n* (%)				0.341
Death	20,284 (67)	131 (64)	20,153 (67)	
Live	9875 (33)	74 (36)	9801 (33)	
Disease‐specific outcome, *n* (%)				0.29
Death from HCC	14,596 (48)	88 (43)	14,508 (48)	
Death from Others	5688 (19)	43 (21)	5645 (19)	
Live	9875 (33)	74 (36)	9801 (33)	
Age, Median (Q1, Q3)	63 (57, 71)	67 (59, 75)	63 (57, 71)	<0.001
Sex, *n* (%)				0.001
Female	6973 (23)	67 (33)	6906 (23)	
Male	23,186 (77)	138 (67)	23,048 (77)	
Race, *n* (%)				0.186
Black	4138 (14)	20 (10)	4118 (14)	
Others	5178 (17)	41 (20)	5137 (17)	
White	20,843 (69)	144 (70)	20,699 (69)	
Marital, *n* (%)				0.055
Divorced/Separated	4352 (14)	20 (10)	4332 (14)	
Married	15,046 (50)	115 (56)	14,931 (50)	
Single/Unmarried	6448 (21)	35 (17)	6413 (21)	
Widowed/Others	4313 (14)	35 (17)	4278 (14)	
Diagnosis, *n* (%)				0.098
2010	4253 (14)	43 (21)	4210 (14)	
2011	4577 (15)	26 (13)	4551 (15)	
2012	4941 (16)	34 (17)	4907 (16)	
2013	5216 (17)	34 (17)	5182 (17)	
2014	5509 (18)	37 (18)	5472 (18)	
2015	5663 (19)	31 (15)	5632 (19)	
AJCC, *n* (%)				0.23
I	13,151 (44)	99 (48)	13,052 (44)	
II	6510 (22)	33 (16)	6477 (22)	
III	5393 (18)	35 (17)	5358 (18)	
IV	5105 (17)	38 (19)	5067 (17)	
AJCC.T, *n* (%)				0.024
T0 ~ T1	14,225 (47)	111 (54)	14,114 (47)	
T2	7148 (24)	36 (18)	7112 (24)	
T3	6925 (23)	39 (19)	6886 (23)	
T4	980 (3)	12 (6)	968 (3)	
TX	881 (3)	7 (3)	874 (3)	
AJCC.N, *n* (%)				0.21
N+	2743 (9)	13 (6)	2730 (9)	
N0	27,416 (91)	192 (94)	27,224 (91)	
AJCC.M, *n* (%)				0.307
M0	26,246 (87)	173 (84)	26,073 (87)	
M1	3913 (13)	32 (16)	3881 (13)	
Mets. bone, *n* (%)				0.468
NO/Unknown	28,917 (96)	194 (95)	28,723 (96)	
YES	1242 (4)	11 (5)	1231 (4)	
Mets. brain, *n* (%)				1
NO/Unknown	30,074 (100)	205 (100)	29,869 (100)	
YES	85 (0)	0 (0)	85 (0)	
Mets. liver, *n* (%)				0.727
NO/Unknown	29,855 (99)	204 (100)	29,651 (99)	
YES	304 (1)	1 (0)	303 (1)	
Mets. lung, *n* (%)				0.113
NO/Unknown	28,732 (95)	190 (93)	28,542 (95)	
YES	1427 (5)	15 (7)	1412 (5)	
Surgery, *n* (%)				<0.001
NO	21,713 (72)	108 (53)	21,605 (72)	
YES	8446 (28)	97 (47)	8349 (28)	
Radiotherapy, *n* (%)				0.06
NO	29,753 (99)	199 (97)	29,554 (99)	
YES	406 (1)	6 (3)	400 (1)	
Chemotherapy, *n* (%)				<0.001
NO	15,691 (52)	134 (65)	15,557 (52)	
YES	14,468 (48)	71 (35)	14,397 (48)	
Is.primary, *n* (%)				<0.001
NO	3832 (13)	44 (21)	3788 (13)	
YES	26,327 (87)	161 (79)	26,166 (87)	
Grade, *n* (%)				<0.001
I	3414 (11)	31 (15)	3383 (11)	
II	5242 (17)	72 (35)	5170 (17)	
III ~ IV	2316 (8)	17 (8)	2299 (8)	
Unknown	19,187 (64)	85 (41)	19,102 (64)	
Age.cat, *n* (%)				<0.001
0 ~ 59	10,706 (35)	60 (29)	10,646 (36)	
60 ~ 64	6495 (22)	27 (13)	6468 (22)	
65 ~ 69	4683 (16)	31 (15)	4652 (16)	
70 ~ 99	8275 (27)	87 (42)	8188 (27)	

Abbreviations: AJCC. T, AJCC tumor staging; AJCC. N, AJCC lymph node staging; AJCC. M, AJCC distant metastasis staging; Mets. bone/brain/liver/lung, distant metastasis to bone/brain/liver/lung from primary HCC; is.primary, is primary discovered cancer; Age.cat, age transformed to categorical variable.

### 3.2 Comparison of survival analysis between patients with HCC‐clear and HCC‐NOS

**FIGURE 2:**
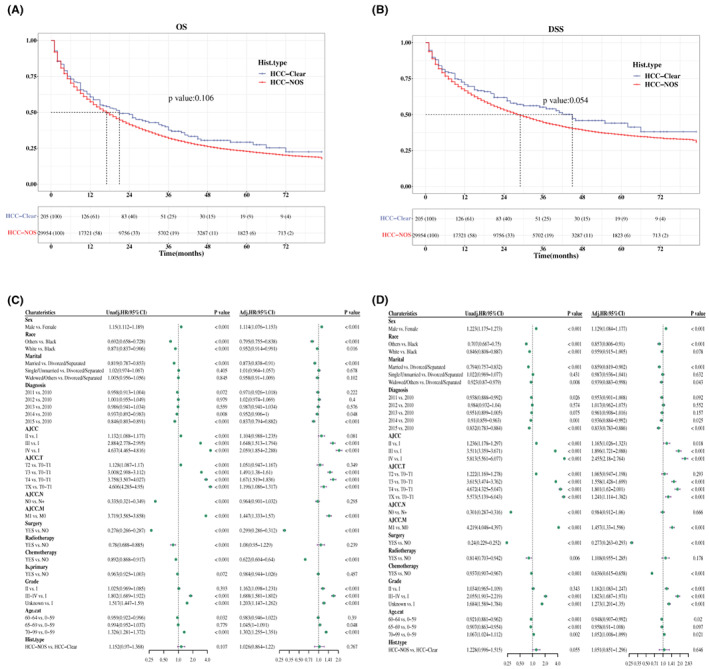
Comparative analysis of survival rate in patients with HCC. (A, B) Comparison of OS (A) and DSS (B) between patients with HCC‐clear and HCC‐NOS. Data shown below the Kaplan–Meier curve were the number at risk in separate histological types. The median survival time was the time corresponding to the survival rate of 50%, presented as a dotted line. (C, D) Analysis of prognostic factors in patients with HCC according to the multivariate Cox proportional‐hazards model for OS (C) and DSS (D) visualized in a forest plot. Adj.HR, adjusted hazard ratio; Age.cat, age transformed to categorical variable; AJCC. M, AJCC distant metastasis staging; AJCC. N, AJCC lymph node staging; AJCC. T, AJCC tumor staging; DSS, disease‐specific survival; Hist.type, histopathological type; is.primary, is primary discovered cancer; OS, overall survival; Unadj.HR, unadjusted hazard ratio.

To better understand the survival difference between the two groups, survival curves for OS and DSS were constructed in patients with certain survival status and time. Summarily, there was no significant difference between patients with HCC‐clear and HCC‐NOS. Specifically, the median OS time in HCC‐clear and HCC‐NOS is 21 (95% CI, 15–33) and 17 (95% CI, 17–18) months, respectively (*p* = 0.106, Figure 2A), while the median DSS time in each group is 45 and 29 months (*p* = 0.054, Figure 2B).

A multivariate Cox regression analysis was performed to access the risk factors involved in the prognosis of patients with HCC‐clear and HCC‐NOS. Among these variables, sex, AJCC cancer staging, AJCC distant metastasis staging, surgical status, chemotherapy, and grade, respectively, were independent prognostic factor of patients with HCC‐clear and HCC‐NOS in OS and DSS analysis (Figure 2C,D). Meanwhile, the histopathological type had no significant impact on OS (adjusted HR, 1.026; 95% CI, 0.864–1.22) and DSS (adjusted HR, 1.051; 95%CI, 0.851–1.296) (*p* > 0.05, Figure 2C,D).

**FIGURE 3:**
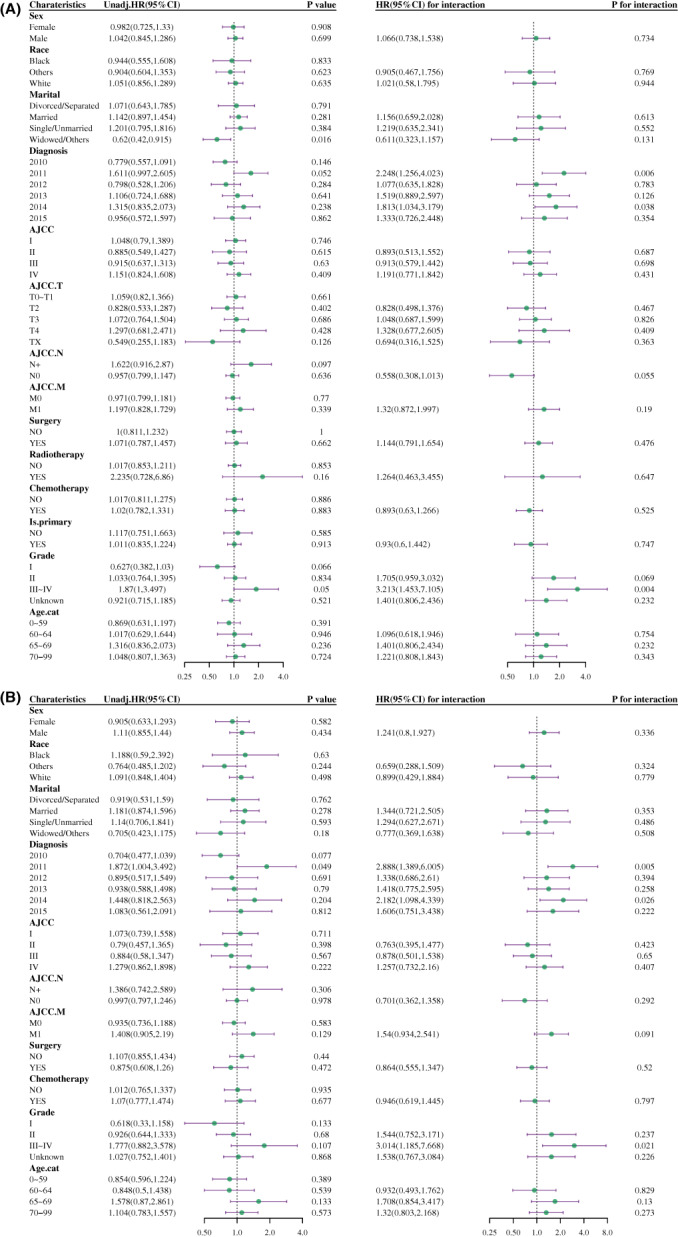
Subgroup analysis and interaction assessment for OS (A) and DSS (B). HCC‐NOS vs. HCC‐clear. In the left panel of subgroup analysis, hazard ratio (HR) represented the mortality rate of patients with HCC‐NOS compared to HCC‐clear in a specific subgroup. *p* < 0.05 was considered statistically significant. In the right panel of interaction assessment, each first subgroup was set as reference, HR for interaction showed the interaction effect on the association of pathological type (HCC‐NOS vs. HCC‐clear) and patient survival in the indicated subgroup compared to the reference group. *p* interaction value <0.05 was considered a statistically significant interaction. Age.cat, age transformed to categorical variable; AJCC. M, AJCC distant metastasis staging; AJCC. N, AJCC lymph node staging; AJCC. T, AJCC tumor staging; DSS, disease‐specific survival; is.primary, is primary discovered cancer; OS, overall survival; Unadj.HR, unadjusted hazard ratio.

Next, we wanted to know whether the histopathological type HCC was correlated with prognosis when related prognostic factors were classified into subgroups. Patients with HCC‐clear had a better survival rate at grade III ~ IV compared to HCC‐NOS (HR, 1.87; 95% CI, 1–3.497, *p* = 0.05, Figure 3A, left panel) in terms of OS analysis, and the diagnosis year of 2011 was associated with worse survival in patients with HCC‐NOS compared to HCC‐clear in DSS analysis (HR, 1.872; 95% CI, 1.004–3.492, *p* = 0.049, Figure 3B, left panel). Moreover, the association between pathological type and OS rate of patients in the grade III ~ IV subgroup was 3.213 times higher than that in grade I (95% CI, 1.453–7.105) (*p* for interaction = 0.004, Figure 3A, right panel) in the interaction assessment. While the relationship between pathological type and DSS rate of patients in the diagnosis year 2011 subgroup was 2.888 (95% CI, 1.389–6.005) times higher than that in 2010 (*p* for interaction = 0.005, Figure 3B, right panel).

### 3.3 Prognostic comparison between patients with HCC‐clear and HCC‐NOS based on competing risk model

**FIGURE 4:**
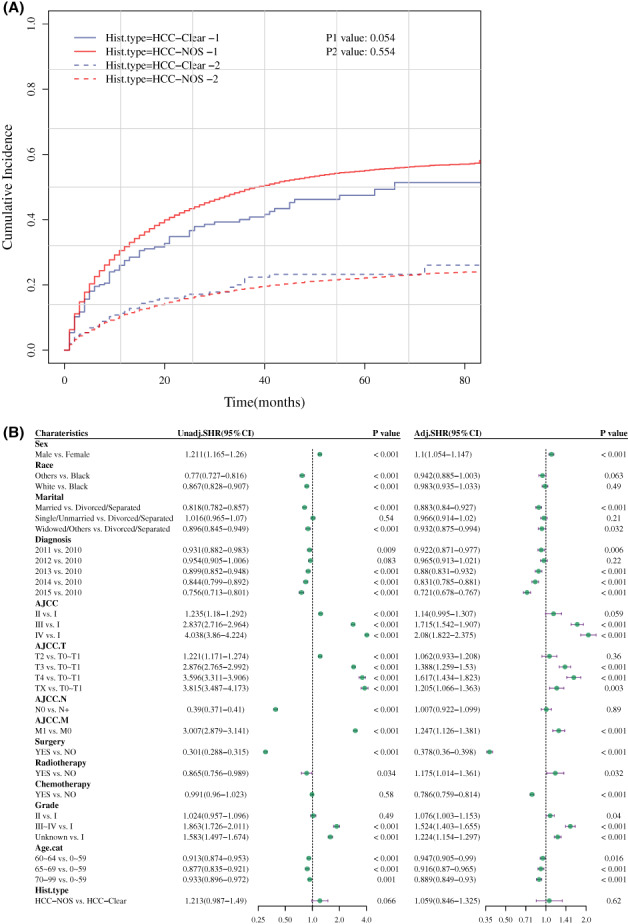
Cumulative incidence function curve and analysis of prognostic factors in patients with HCC based on competing risk model. (A) Cumulative incidence function curve of patients with HCC‐clear and HCC‐NOS for death due to cancer (P1) shown as a solid line and death from other causes (P2) shown as a dotted line. (B) Identification of prognostic factors in patients with HCC based on competing risk model. *p* < 0.05 was considered statistically significant. Adj.SHR, adjusted sub‐distribution hazard ratio; Age.cat, age transformed to categorical variable; AJCC. M, AJCC distant metastasis staging; AJCC. N, AJCC lymph node staging; AJCC. T, AJCC tumor staging; Hist.type, histopathological type; is.primary, is primary discovered cancer; Unadj.SHR, unadjusted sub‐distribution hazard ratio.

Considering the competing risk events, the cumulative incidence function curve was plotted based on the competing risk model. Death specifically from HCC and death not specifically from HCC were not statistically different between the two groups (*p* = 0.054 and *p* = 0.554, respectively, Figure 4A). The type of histopathology was also not related to death due to HCC (adjusted SHR, 1.059; 95% CI, 0.846–1.325) (*p* > 0.05, Figure 4B). Subsequently, these data were further validated by the multivariate Cox regression analysis based on competing risk model from sensitivity dataset (adjusted HR, 0.967; 95% CI, 0.851–1.099, *p* > 0.05, Figure S1B).

### 3.4 PSM based on histopathological type

Furthermore, the standard mean differences (SMD) were measured in each covariate to examine the potential confounders involving the comparison between patients with HCC‐clear and HCC‐NOS (Table 2). Since SMD values of almost all covariates were greater than 0.1, PSM was executed according to histopathological type to present the difference more objectively between the two groups. After applying the PSM method, all covariates were well balanced between the two study groups (Table 2 and Figure S2). Furthermore, 205 patients with HCC‐clear were matched with 410 patients with HCC‐NOS for the following survival analysis.

**TABLE 2:** Characteristics of censored population before and after PSM.

	Unmatched					Matched				
Variables		HCC‐Clear	HCC‐NOS	*p*	SMD		HCC‐Clear	HCC‐NOS	*p*	SMD
*n*		205	29,954				205	410		
Sex (%)	Female	67 (32.7)	6906 (23.1)	0.001	0.216	Female	67 (32.7)	118 (28.8)	0.367	0.085
	Male	138 (67.3)	23,048 (76.9)			Male	138 (67.3)	292 (71.2)		
Race (%)	Black	20 (9.8)	4118 (13.7)	0.186	0.135	Black	20 (9.8)	41 (10.0)	0.843	0.05
	Others	41 (20.0)	5137 (17.1)			Others	41 (20.0)	74 (18.0)		
	White	144 (70.2)	20,699 (69.1)			White	144 (70.2)	295 (72.0)		
Marital (%)	Divorced/Separated	20 (9.8)	4332 (14.5)	0.055	0.202	Divorced/Separated	20 (9.8)	36 (8.8)	0.898	0.066
	Married	115 (56.1)	14,931 (49.8)			Married	115 (56.1)	222 (54.1)		
	Single/Unmarried	35 (17.1)	6413 (21.4)			Single/Unmarried	35 (17.1)	74 (18.0)		
	Widowed/Others	35 (17.1)	4278 (14.3)			Widowed/Others	35 (17.1)	78 (19.0)		
Diagnosis (%)	2010	43 (21.0)	4210 (14.1)	0.098	0.202	2010	43 (21.0)	78 (19.0)	0.868	0.117
	2011	26 (12.7)	4551 (15.2)			2011	26 (12.7)	53 (12.9)		
	2012	34 (16.6)	4907 (16.4)			2012	34 (16.6)	75 (18.3)		
	2013	34 (16.6)	5182 (17.3)			2013	34 (16.6)	57 (13.9)		
	2014	37 (18.0)	5472 (18.3)			2014	37 (18.0)	87 (21.2)		
	2015	31 (15.1)	5632 (18.8)			2015	31 (15.1)	60 (14.6)		
AJCC (%)	I	99 (48.3)	13,052 (43.6)	0.23	0.152	I	99 (48.3)	211 (51.5)	0.862	0.074
	II	33 (16.1)	6477 (21.6)			II	33 (16.1)	58 (14.1)		
	III	35 (17.1)	5358 (17.9)			III	35 (17.1)	65 (15.9)		
	IV	38 (18.5)	5067 (16.9)			IV	38 (18.5)	76 (18.5)		
AJCC.T (%)	T0 ~ T1	111 (54.1)	14,114 (47.1)	0.024	0.229	T0 ~ T1	111 (54.1)	236 (57.6)	0.932	0.078
	T2	36 (17.6)	7112 (23.7)			T2	36 (17.6)	66 (16.1)		
	T3	39 (19.0)	6886 (23.0)			T3	39 (19.0)	69 (16.8)		
	T4	12 (5.9)	968 (3.2)			T4	12 (5.9)	25 (6.1)		
	TX	7 (3.4)	874 (2.9)			TX	7 (3.4)	14 (3.4)		
AJCC.N (%)	N+	13 (6.3)	2730 (9.1)	0.21	0.104	N+	13 (6.3)	29 (7.1)	0.865	0.029
	N0	192 (93.7)	27,224 (90.9)			N0	192 (93.7)	381 (92.9)		
AJCC.M (%)	M0	173 (84.4)	26,073 (87.0)	0.307	0.076	M0	173 (84.4)	343 (83.7)	0.907	0.02
	M1	32 (15.6)	3881 (13.0)			M1	32 (15.6)	67 (16.3)		
Surgery (%)	NO	108 (52.7)	21,605 (72.1)	<0.001	0.41	NO	108 (52.7)	211 (51.5)	0.842	0.024
	YES	97 (47.3)	8349 (27.9)			YES	97 (47.3)	199 (48.5)		
Radiotherapy (%)	NO	199 (97.1)	29,554 (98.7)	0.096	0.11	NO	199 (97.1)	401 (97.8)	0.782	0.046
	YES	6 (2.9)	400 (1.3)			YES	6 (2.9)	9 (2.2)		
Chemotherapy (%)	NO	134 (65.4)	15,557 (51.9)	<0.001	0.275	NO	134 (65.4)	266 (64.9)	0.976	0.01
	YES	71 (34.6)	14,397 (48.1)			YES	71 (34.6)	144 (35.1)		
Is.primary (%)	NO	44 (21.5)	3788 (12.6)	<0.001	0.236	NO	44 (21.5)	84 (20.5)	0.861	0.024
	YES	161 (78.5)	26,166 (87.4)			YES	161 (78.5)	326 (79.5)		
Grade (%)	I	31 (15.1)	3383 (11.3)	<0.001	0.492	I	31 (15.1)	69 (16.8)	0.961	0.047
	II	72 (35.1)	5170 (17.3)			II	72 (35.1)	142 (34.6)		
	III ~ IV	17 (8.3)	2299 (7.7)			III ~ IV	17 (8.3)	33 (8.0)		
	Unknown	85 (41.5)	19,102 (63.8)			Unknown	85 (41.5)	166 (40.5)		
Age.cat (%)	0 ~ 59	60 (29.3)	10,646 (35.5)	<0.001	0.349	0 ~ 59	60 (29.3)	118 (28.8)	0.863	0.073
	60 ~ 64	27 (13.2)	6468 (21.6)			60 ~ 64	27 (13.2)	45 (11.0)		
	65 ~ 69	31 (15.1)	4652 (15.5)			65 ~ 69	31 (15.1)	64 (15.6)		
	70–99	87 (42.4)	8188 (27.3)			70–99	87 (42.4)	183 (44.6)		

Abbreviations: AJCC. T, AJCC tumor staging; AJCC. N, AJCC lymph node staging; AJCC. M, AJCC distant metastasis staging; is.primary, is primary discovered cancer; Age.cat, age transformed to categorical variable.

### 3.5 Comparison of survival analysis between patients with HCC‐clear and HCC‐NOS after PSM

**FIGURE 5:**
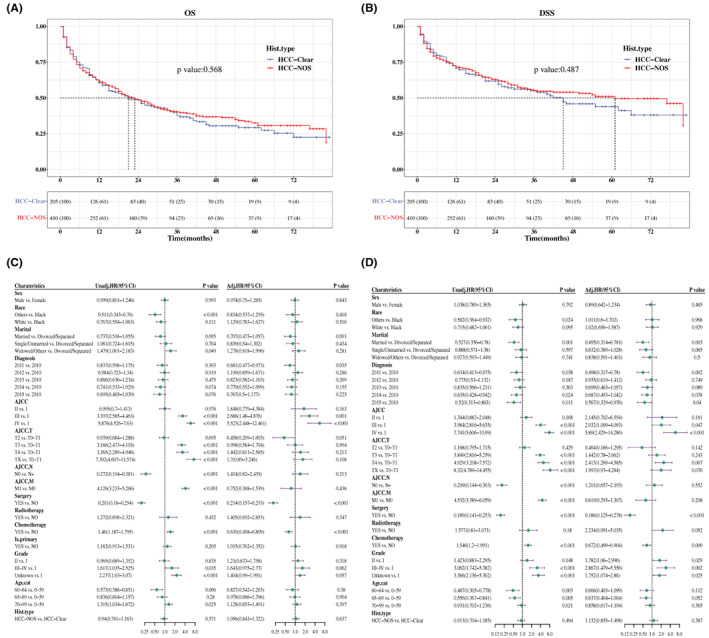
Survival analysis after PSM. (A, B) Comparison of OS (A) and DSS (B) between patients with HCC‐clear and HCC‐NOS. Data shown below the Kaplan–Meier curve were the number at risk in separate histological type. The median survival time was the time corresponding to survival rate of 50%, presented as a dotted line. (C, D) Multivariate regression analysis for OS (C) and DSS (D). Adj.HR, adjusted hazard ratio; AJCC. T, AJCC tumor staging; Age.cat, age transformed to categorical variable; AJCC. M, AJCC distant metastasis staging; AJCC. N, AJCC lymph node staging; DSS, disease‐specific survival; Hist.type, histopathological type; is.primary, is primary discovered cancer; OS, overall survival; Unadj.HR, unadjusted hazard ratio.

In the comparison of matched groups after PSM, no significant differences were observed in survival rate for OS or DSS (*p* > 0.05, Figure 5A,B). In the multivariate Cox regression analysis, the diagnosis year of 2011 versus 2010, AJCC staging IV versus I, surgical status, and chemotherapy, respectively, still represented independent risk factors of patients with HCC‐clear and HCC‐NOS in OS and DSS analysis (Figure 5C,D). However, in consistently with results before PSM, the histopathological type was not associated with higher or lower risk after adjusting for potential confounding factors (Figure 5C,D).

**FIGURE 6:**
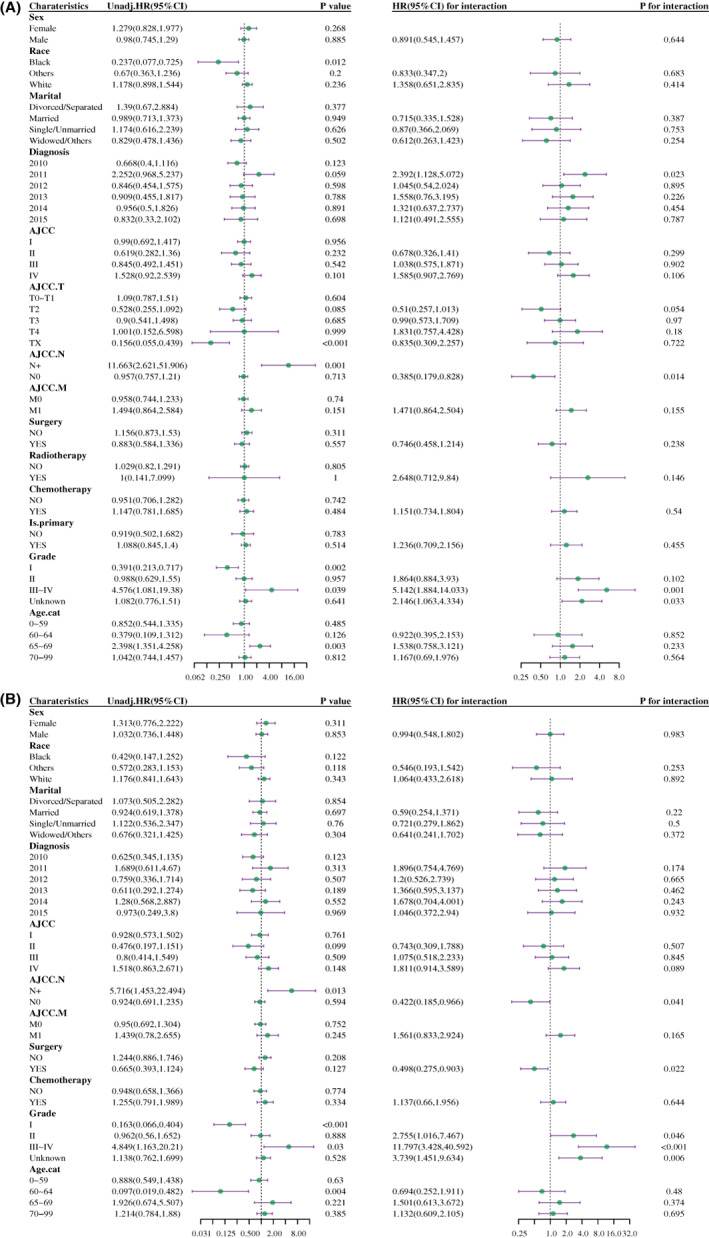
Subgroup analysis and interaction assessment for OS (A) and DSS (B) after PSM. HCC‐NOS vs. HCC‐clear. In the left panel of subgroup analysis, hazard ratio (HR) represented the mortality rate of patients with HCC‐NOS compared to HCC‐clear in a specific subgroup. *p* < 0.05 was considered statistically significant. In the right panel of interaction assessment, each first subgroup was set as reference, HR for interaction showed the interaction effect on the association of pathological type (HCC‐NOS vs. HCC‐clear) and patient survival in the indicated subgroup compared to the reference group. *p* interaction value <0.05 was considered a statistically significant interaction. Age.cat, age transformed to categorical variable; AJCC. M, AJCC distant metastasis staging; AJCC. N, AJCC lymph node staging; AJCC. T, AJCC tumor staging; DSS, disease‐specific survival; is.primary, is primary discovered cancer; OS, overall survival; Unadj.HR, unadjusted hazard ratio.

Interestingly, the prognostic factors that interacted with histopathological type after PSM in the subgroup were inconsistent with the analysis before matching. Patients with HCC‐clear had a better survival rate with lymph node metastasis (HR, 11.663; 95% CI, 2.621–51.906, *p* = 0.001, Figure 6A, left panel) and if tumor was in grade III ~ IV (HR, 4.576; 95% CI, 1.081–19.38, *p* = 0.039, Figure 6A, left panel) compared to patients with HCC‐NOS for OS analysis. Moreover, the association between pathological type and OS rate of patients without lymph node metastasis was 0.385 times lesser than those with lymph node metastasis (95% CI, 0.179–0.828) (*p* for interaction = 0.014, Figure 6A, right panel), while this ratio in the grade III ~ IV subgroup was 5.142 (95% CI, 1.884–14.033) times higher than that in the grade I (*p* for interaction = 0.001, Figure 6A, right panel) in the interaction assessment. The result of DSS in prognostic analysis after PSM was similar to OS (Figure 6B).

### 3.6 Prognostic comparison between patients with HCC‐clear and HCC‐NOS based on competing risk model after PSM

**FIGURE 7:**
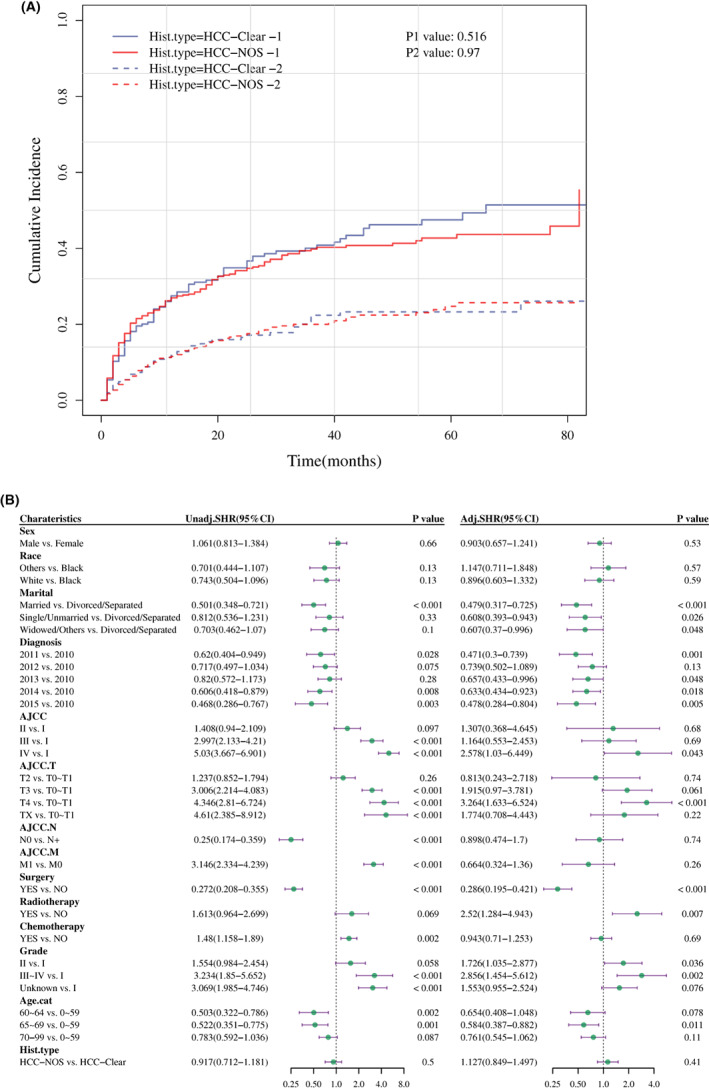
Cumulative incidence function curve and prognostic analysis based on competing risk model after PSM. (A) Cumulative incidence function curve of patients with HCC‐clear and HCC‐NOS for death due to cancer (P1) presented as a solid line, and death from other causes (P2) shown as a dotted line. (B) Analysis of prognostic factors in patients with HCC based on competing risk model. Adj.SHR, adjusted sub‐distribution hazard ratio; AJCC. T, AJCC tumor staging; Age.cat, age transformed to categorical variable; AJCC. M, AJCC distant metastasis staging; AJCC. N, AJCC lymph node staging; Hist.type, histopathological type; is.primary, is primary discovered cancer; Unadj.SHR, unadjusted sub‐distribution hazard ratio.

The cumulative incidence was calculated after PSM based on the competing risk model. There were no significant differences between the two groups from death due to cancer and other causes of death (*p* > 0.05, Figure 7A). The histopathological type was also not related to the death due to HCC (adjusted SHR, 1.127; 95% CI, 0.849–1.497) (*p* > 0.05, Figure 7B).

## 4 DISCUSSION

HCC‐clear is rare neoplasm with undetermined survival and prognostic factors. Evaluating the prognosis of HCC‐clear may help doctors establish and implement appropriate regimens which have not yet achieved general consensus. In this investigation, significant demographic and clinicopathological characteristics were used to explore the difference between HCC‐clear and HCC‐NOS based on data from the SEER database. OS and DSS were utilized to reflect that histopathological type was not a prognostic factor in the two groups. Additionally, applying the competing risk model showed that the histopathological type was not bound to the mortality rate due to HCC. The conclusion obtained after PSM and sensitivity analysis were performed was similar.

A total of 205 cases of patients with HCC‐clear from the SEER database enrolled in our study according to the inclusion and exclusion criteria accounted for nearly 0.68% of all HCC cases, in agreement with previous reports.^6^, ^8^ Our study discovered that patients with HCC‐clear were generally older, most often 70–99, and were predominantly female compared to HCC‐NOS, which was in accordance with a previous study though the difference found in that study was not significant.^25^ More patients with HCC‐clear were divided into AJCC tumor staging T0–T1 and T4, as other research teams have previously shown using data from SEER the database. Often there is a tendency to the absence of past cancer history, which was not reported previously.^26^

Surgical treatment was thought to be the best procedure for HCC‐clear, it had many advantages, such as tumor mass reduction, improved long‐term survival, and lower recurrence and metastasis.^12^, ^14^, ^27^, ^28^ Indeed, we found nearly half the population of patients with HCC‐clear would choose surgery as the primary treatment method, while chemotherapy was the first choice for more patients with HCC‐NOS. Nevertheless, hepatic resection is not the standard treatment for patients with HCC. However, in the recurrence population of patients with HCC‐clear, the re‐resection group had a longer survival time than other therapeutic methods; there was no significant difference for OS.^29^ In our study, surgery and chemotherapy were all independent prognostic factors for patients with HCC‐clear and HCC‐NOS, but in the subgroup analysis, these two types of HCC did not show a prognostic difference. The number of subtypes of HCC is limited. Hence, for the effective treatment of subtype and common type HCC, especially occurrence of recurrence, adjuvant therapies warrant further investigation.

Currently, there is wide controversy and not up‐to‐date prognostic comparison between HCC‐clear and HCC‐NOS. Tao et al. discovered that the 1‐, 3‐, and 5‐year OS rates were much better in HCC‐clear than HCC‐NOS and the recurrence rate was lower in patients with HCC‐clear.^29^ Li et al. found that HCC‐clear tends to be a lower malignancy and better prognosis subtype HCC.^30^ Nevertheless, Yang et al. concluded that the prognosis of clear cell type HCC was similar to normal type by comparing 20 cases of clear cell HCC and 118 cases of non‐clear cell HCC.^11^ In recent research, OS was comparable for patients with HCC‐clear and HCC‐NOS.^26^ In addition, two teams studied the prognosis of patients after hepatectomy and found that patients with HCC‐clear showed a favorable postoperative prognosis than those with the common type.^12^, ^27^ However, most studies were limited by the small number of cases due to the rarity of HCC‐clear or were not analyzed in detail. Furthermore, some studies did not take the cancer‐specific survival and confounding factors into account. In our investigation, not only OS but also DSS were focused on. To avoid the competing risk of other causes of death, a competing risk model was introduced to evaluate a more accurate prognosis of different histopathological types.^31^, ^32^ Based on our analysis, histopathological type was not a prognostic factor affecting OS, DSS, and cumulative incidence of cancer‐specific death when comparing HCC‐clear and HCC‐NOS. The survival outcomes were confirmed by applying PSM and sensitivity analysis.

In accordance with most of the available literature, HCC‐clear is a low‐grade malignancy neoplasm.^14^, ^33^ In a stepwise manner, we examined more detailed prognostic information that previous investigators did not focus on. Whether tumor grade and lymph node were independent prognostic factors of HCC‐clear or not lacked sufficient data to be categorized.^14^, ^26^ However, in the subgroup analysis, we uncovered that patients with HCC‐clear had improved prognosis for OS and DSS when Edmondson's tumor grade was III ~ IV after PSM, especially compared to grade I in interaction assessment. Moreover, in patients with HCC‐clear with lymph node metastasis, better OS and DSS were observed after PSM adjustment for confounding factors. These results give hepatic surgeons a more precise prognosis for different types of patients so that they can provide specific regimens according to tumor grade and metastasis of lymph nodes.

To the best of our knowledge, this is the first work comprising 30,159 cases into HCC‐clear and HCC‐NOS comparative analysis and 67,373 cases into sensitivity analysis to afford statistical robustness. Indeed, a competing risk model is a better way to evaluate cancer‐specific survival more precisely by excluding unknown confounding factors. Finally, our results were finessed through sensitivity analysis on different datasets. Nevertheless, our work had some limitations. Firstly, since the SEER database only recruits American people, the clinical relevance for other races could not be extrapolated. Secondly, the variables were limited in the SEER database, and some important clinical information was not available or shown as *No* or *Unknown*. In future research, we could combine multicenter studies in different regions to obtain a more solid conclusion.

The current study provides the convincing statistical evidence on the demographic and clinicopathological characteristics and detailed prognosis based on a large population study for patients with HCC‐clear and HCC‐NOS, thereby helping hepatic surgeons to make better‐informed judgments on patient care.

## AUTHOR CONTRIBUTIONS

XZ performed the computational analysis and wrote the paper; XGF and XWH designed the experiments and edited the paper. All authors have approved the final manuscript.

## CONFLICT OF INTEREST STATEMENT

The authors declare no competing interests.

## ETHICS APPROVAL

This article does not contain any studies with human participants or animals performed by any of the authors.

## Supporting information


Figure S1.

Figure S2.

## Data Availability

The data that support the findings of this study are available from the corresponding author upon reasonable request.
