# Supplementary material for: Competing risk model for prognostic comparison between clear cell type and common type hepatocellular carcinoma: A population‐based propensity score matching study
Source: Cancer Med. 2023 Mar 19;12(9):10406–22. doi: 10.1002/cam4.5773 (PMC10225237; doi:10.1002/cam4.5773)
Supplement: Supplementary file 1 — Figure S1. [file CAM4-12-10406-s002.pdf]

A.

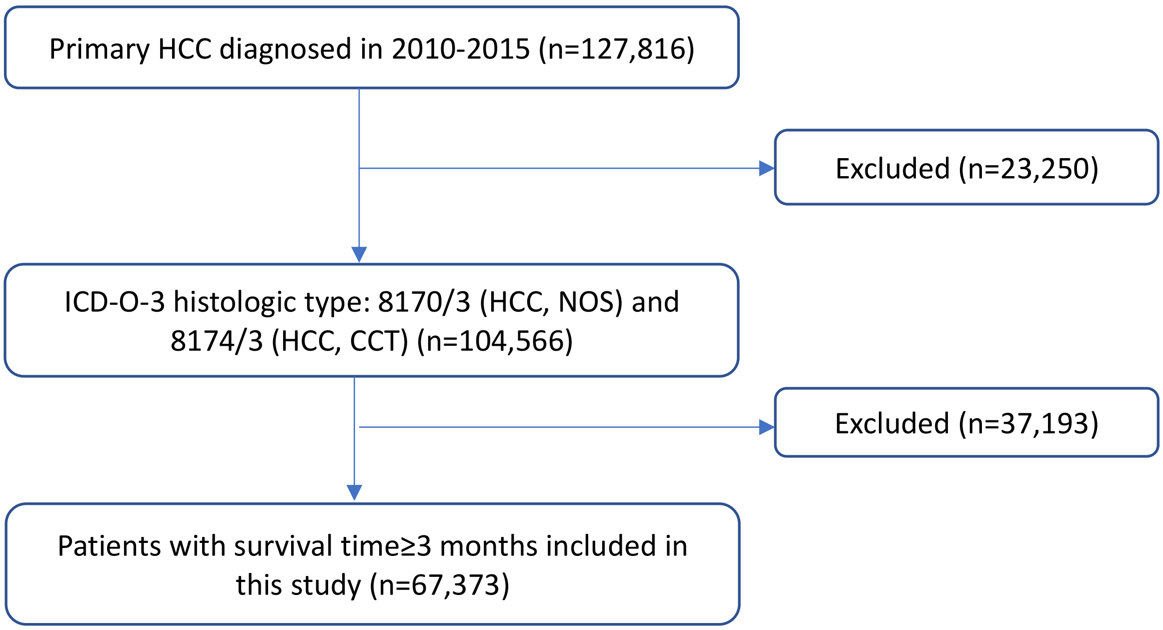

Primary HCC diagnosed in 2010-2015 (n=127,816)
Excluded (n=23,250)
ICD-O-3 histologic type: 8170/3 (HCC, NOS) and 8174/3 (HCC, CCT) (n=104,566)
Excluded (n=37,193)
Patients with survival time≥3 months included in this study (n=67,373)

B.

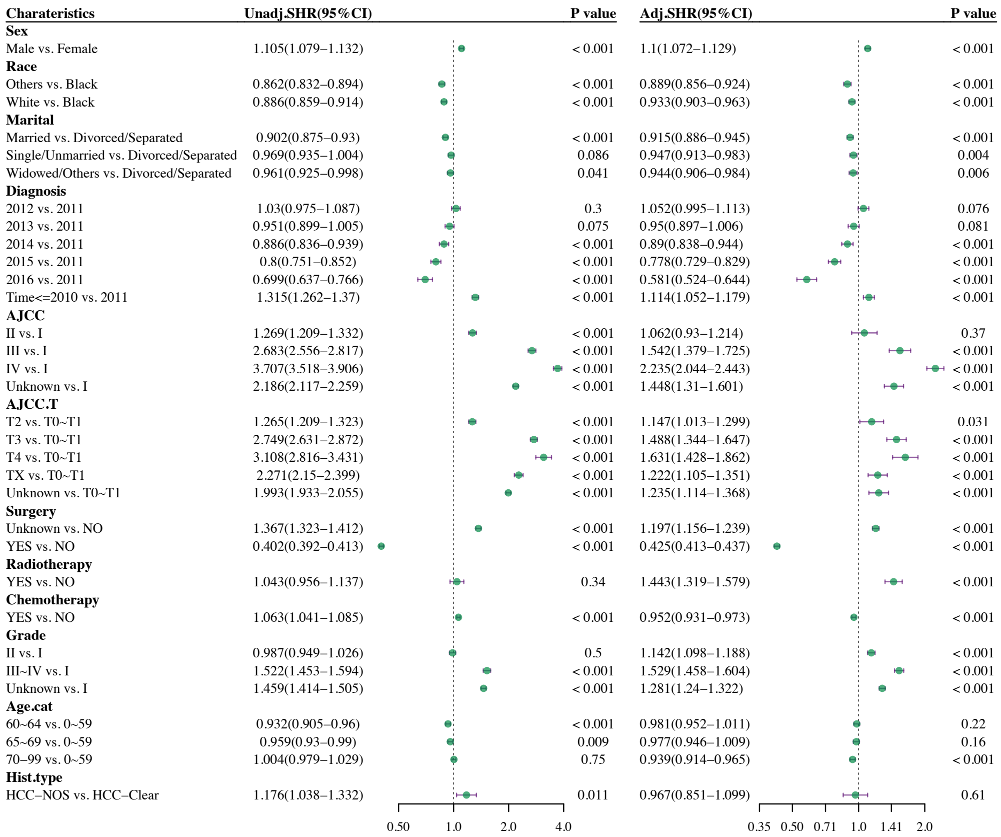

Charateristics | Unadj.SHR(95%CI) | P value | Adj.SHR(95%CI) | P value
Sex
Male vs. Female | 1.105(1.079–1.132) | < 0.001 | 1.1(1.072–1.129) | < 0.001
Race
Others vs. Black | 0.862(0.832–0.894) | < 0.001 | 0.889(0.856–0.924) | < 0.001
White vs. Black | 0.886(0.859–0.914) | < 0.001 | 0.933(0.903–0.963) | < 0.001
Marital
Married vs. Divorced/Separated | 0.902(0.875–0.93) | < 0.001 | 0.915(0.886–0.945) | < 0.001
Single/Unmarried vs. Divorced/Separated | 0.969(0.935–1.004) | 0.086 | 0.947(0.913–0.983) | 0.004
Widowed/Others vs. Divorced/Separated | 0.961(0.925–0.998) | 0.041 | 0.944(0.906–0.984) | 0.006
Diagnosis
2012 vs. 2011 | 1.03(0.975–1.087) | 0.3 | 1.052(0.995–1.113) | 0.076
2013 vs. 2011 | 0.951(0.899–1.005) | 0.075 | 0.95(0.897–1.006) | 0.081
2014 vs. 2011 | 0.886(0.836–0.939) | < 0.001 | 0.89(0.838–0.944) | < 0.001
2015 vs. 2011 | 0.8(0.751–0.852) | < 0.001 | 0.778(0.729–0.829) | < 0.001
2016 vs. 2011 | 0.699(0.637–0.766) | < 0.001 | 0.581(0.524–0.644) | < 0.001
Time<=2010 vs. 2011 | 1.315(1.262–1.37) | < 0.001 | 1.114(1.052–1.179) | < 0.001
AJCC
II vs. I | 1.269(1.209–1.332) | < 0.001 | 1.062(0.93–1.214) | 0.37
III vs. I | 2.683(2.556–2.817) | < 0.001 | 1.542(1.379–1.725) | < 0.001
IV vs. I | 3.707(3.518–3.906) | < 0.001 | 2.235(2.044–2.443) | < 0.001
Unknown vs. I | 2.186(2.117–2.259) | < 0.001 | 1.448(1.31–1.601) | < 0.001
AJCC.T
T2 vs. T0~T1 | 1.265(1.209–1.323) | < 0.001 | 1.147(1.013–1.299) | 0.031
T3 vs. T0~T1 | 2.749(2.631–2.872) | < 0.001 | 1.488(1.344–1.647) | < 0.001
T4 vs. T0~T1 | 3.108(2.816–3.431) | < 0.001 | 1.631(1.428–1.862) | < 0.001
TX vs. T0~T1 | 2.271(2.15–2.399) | < 0.001 | 1.222(1.105–1.351) | < 0.001
Unknown vs. T0~T1 | 1.993(1.933–2.055) | < 0.001 | 1.235(1.114–1.368) | < 0.001
Surgery
Unknown vs. NO | 1.367(1.323–1.412) | < 0.001 | 1.197(1.156–1.239) | < 0.001
YES vs. NO | 0.402(0.392–0.413) | < 0.001 | 0.425(0.413–0.437) | < 0.001
Radiotherapy
YES vs. NO | 1.043(0.956–1.137) | 0.34 | 1.443(1.319–1.579) | < 0.001
Chemotherapy
YES vs. NO | 1.063(1.041–1.085) | < 0.001 | 0.952(0.931–0.973) | < 0.001
Grade
II vs. I | 0.987(0.949–1.026) | 0.5 | 1.142(1.098–1.188) | < 0.001
III~IV vs. I | 1.522(1.453–1.594) | < 0.001 | 1.529(1.458–1.604) | < 0.001
Unknown vs. I | 1.459(1.414–1.505) | < 0.001 | 1.281(1.24–1.322) | < 0.001
Age.cat
60~64 vs. 0~59 | 0.932(0.905–0.96) | < 0.001 | 0.981(0.952–1.011) | 0.22
65~69 vs. 0~59 | 0.959(0.93–0.99) | 0.009 | 0.977(0.946–1.009) | 0.16
70~99 vs. 0~59 | 1.004(0.979–1.029) | 0.75 | 0.939(0.914–0.965) | < 0.001
Hist.type
HCC–NOS vs. HCC–Clear | 1.176(1.038–1.332) | 0.011 | 0.967(0.851–1.099) | 0.61
0.50 1.0 2.0 4.0
0.35 0.50 0.71 1.0 1.41 2.0
