# Supplementary figures and images for: Competing risk model for prognostic comparison between clear cell type and common type hepatocellular carcinoma: A population‐based propensity score matching study
Source: Cancer Med. 2023 Mar 19;12(9):10406–22. doi: 10.1002/cam4.5773 (PMC10225237; doi:10.1002/cam4.5773)

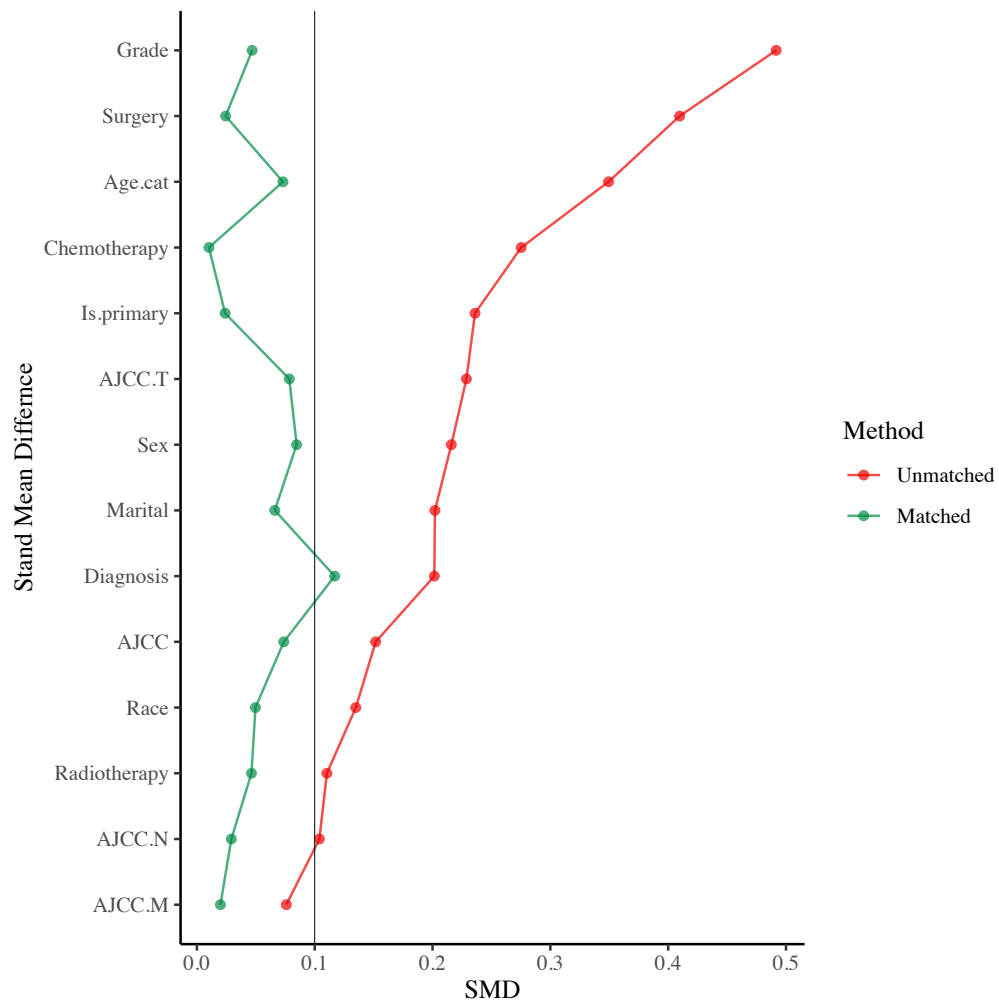

Supplement: Supplementary file 2 — Figure S2. [file CAM4-12-10406-s001.pdf]
